# Bilateral cervical chondrocutaneous branchial remnants: A case report and a review of the literature

**DOI:** 10.1097/MD.0000000000021114

**Published:** 2020-07-10

**Authors:** Han Shin Lee, Tae Han Kim, Jae Yool Jang, Jung Woo Woo, JinKwon Lee, Sang Ho Jeong, Eun Jung Jung, Hyo Jung An, Taejin Park

**Affiliations:** a Department of Surgery; b Department of Pathology, Gyeongsang National University Changwon Hospital, Gyeongsang National University School of Medicine, Changwon, Korea.

**Keywords:** branchial remnant, chondrocutaneous, neck, skin tag

## Abstract

**Rationale:**

Cervical chondrocutaneous branchial remnants are rare, benign, congenital anomalies, frequently seen bilaterally.

**Patient concerns:**

Here, we report the case of a 4-month-old female infant who presented with bilateral lower neck skin tag since birth.

**Diagnosis and Interventions:**

The patient underwent mass excision. The final pathological diagnosis was bilateral cervical chondrocutaneous branchial remnants with hyaline cartilage.

**Outcomes:**

No complications were observed after excision. One-year follow-up revealed no recurrence.

**Lessons:**

Bilateral chondrocutaneous branchial remnants are rare anomalies. They are often associated with cardiac or genitourinary abnormalities. Therefore, additional preoperative imaging of the abdomen and heart are recommended.

## 1 Introduction

Cervical mass is a relatively common pathological condition in the neonatal period. Neonatal neck masses are mostly congenital malformations that occur during transformation into adult derivatives, so branching malformations often occur due to the persistence of the part of the branching device, which should generally disappear.^[1]^ First reported in 1858, benign neck tumors, formerly called “cervical skin tags,” “accessory tragus,” “wattle,” and “cervical auricle,” were retermed “chondrocutaneous branchial remnants (CCBR)” by Altan et al in 1997.^[2,3]^ Generally, CCBRs appear unilaterally or bilaterally. To date, 117 cases have been reported (34 with bilateral lesions) in medical literature.^[4]^ Bilateral CCBR are rare, and multiple differential diagnoses should be considered while diagnosing it according to the location. Herein, we present a case of bilateral CCBR presenting as a neonatal neck mass at the sternocleidomastoid (SCM) muscle level and investigated previous medical literatures about bilateral CCBR case reports (Table 1).

**Table 1:**
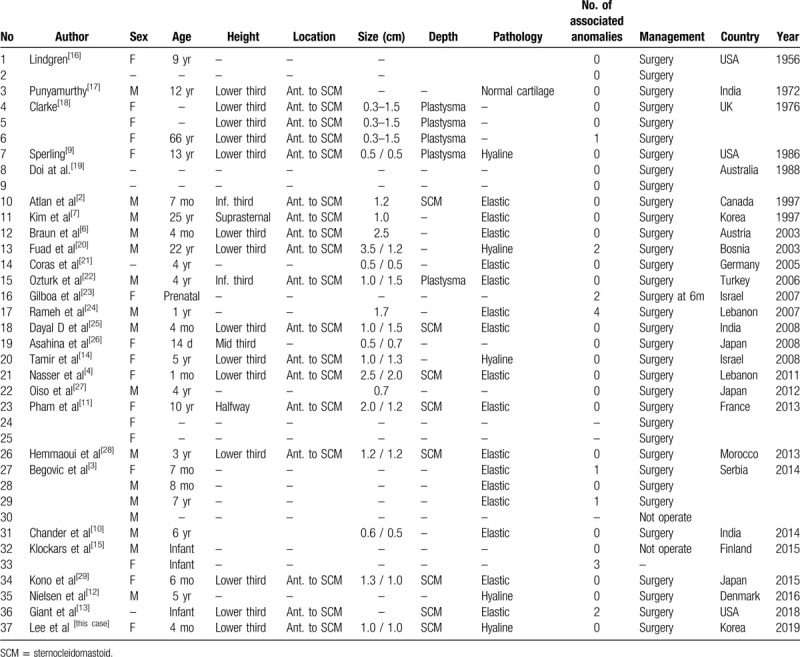
Case reports of bilateral cervical chondrocutaneous branchial remnants.

## 2 Methods

Because this case report is not a prospective or retrospective study, the consent of the patient's parents was sufficient, and ethical approval was provided done by the IRB. Thus, we decided to publish only the age, image findings, and pathologic pictures in the case report, and we received written consent from the patient's parents.

## 3 Case report

### 3.1 Clinical summary

**Figure 1:**
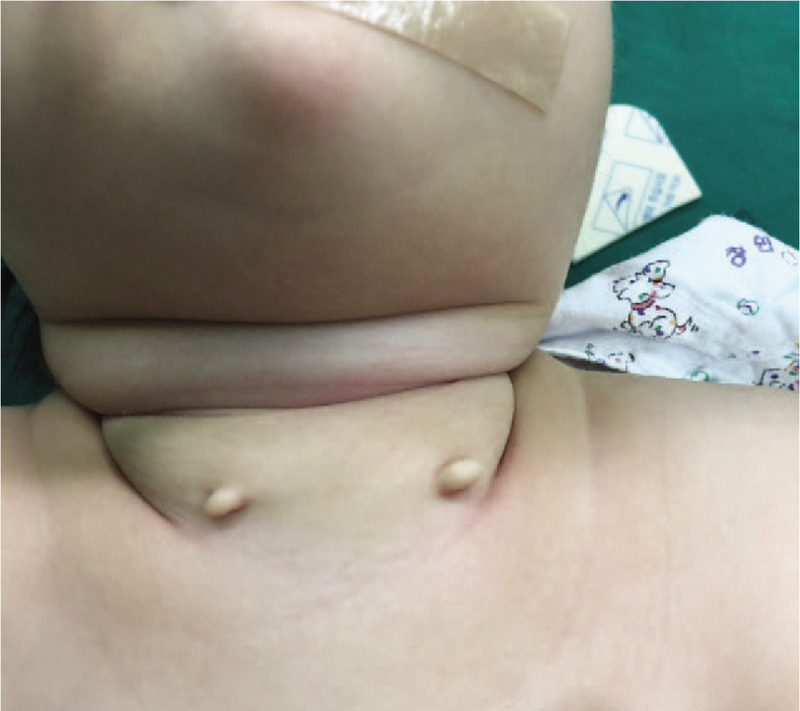
A 4-month-old girl gross findings of bilateral chondrocutaneous branchial remnants (CCBR).

**Figure 2:**
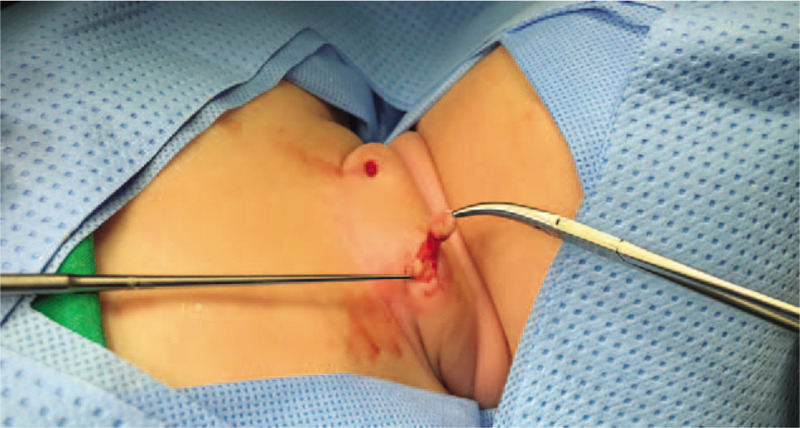
Intraoperative picture of bilateral chondrocutaneous branchial remnants.

A 4-month-old girl was referred to our hospital because of bilateral neck skin lesions since birth. The skin lesions on the neck were covered with normal skin and each lesion measured 1 cm in length (Fig. 1). The lesions were located in the lower third of the neck, anterior to the SCM muscle. The lesions were stiff and elastic. There were no opening pits, discharge, or inflammatory changes. Physical exam was unremarkable. There were no cardiac or urogenital anomalies on ultrasonography. There was no family history of this condition. After obtaining the consent of the parents, surgical excision under general anesthesia was performed. Cartilaginous remnants extended to the fascia of the SCM muscles. There was no fistula tract to deep neck structures (Fig. 2). After mass excision, no recurrence or complications were seen at 1-year follow-up.

### 3.2 Pathological findings

**Figure 3:**
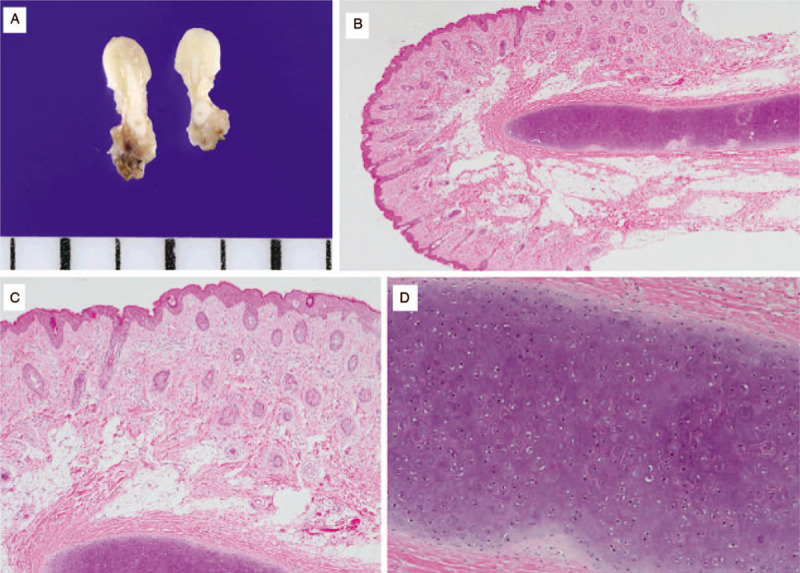
Gross finding and histopathologic findings of excised bilateral chondrocutaneous branchial remnants. (a) The gross finding of excised and cut in half of bilateral chondrocutaneous branchial remnants. The whitish glistening cartilaginous mass is covered by normal skin tissue. (b) On microscope, a polypoid skin lesion with underlying subcutaneous tissue and hyaline cartilage. (c) The overlying skin is composed of many hair follicles, dermal collagen, and adipose tissue. (d) On higher magnification, extracellular matrix of hyaline cartilage and evenly distributed bland-looking chondrocytes.

Histologic examination showed hyaline cartilage cores covered by normal skin consisting of epidermis and dermis with subcutaneous fat compatible with CCBR (Fig. 3).

## 4 Discussion

Neonatal tumors in cervical area are referred when tumors present before the 28th day of life.^[5]^ Tumor located in a newborn's neck includes, differential diagnosis of teratoma, embryoma, hamartoma, dermoid cyst and choristoma.^[6]^ CCBRs are choristomas of the cervical area, and 2 suggestions have been proposed for the embryologic source, although these are yet to be verified. One theory suggests that they arise from ectopic auricular tissue.^[7]^ The other suggests that CCBRs originated from the branchial tissues contributing to the formation of most cervical tissues.^[8,9]^ The core of CCBR is either elastic cartilage or hyaline cartilage. The presence of elastic cartilage may suggest an auricular origin from the first or second branchial arch, whereas the presence of hyaline cartilage excludes an auricular origin and suggests a cervical origin from the second or lower branchial arches.^[9]^ The widely accepted theory of origin is that CCBRs are the result of incomplete obliteration of the branchial apparatus, leaving cells behind in the neck during embryonic migration that differentiate into cartilage. Others suggest that it is rather the presence of pluripotent cell rests, much like the presence of supernumerary nipples, which proliferate into cartilage.^[10]^ These lesions are similar or analogous to preauricular tags, but are located in the lower neck. Most lesions present unilaterally; bilateral lesions as seen in our case are extremely rare.^[3,11]^

In 1997 Altan et al described CCBR as follows:

(1) predominance in male (11 of 17);
(2) high incidence of associated anomalies (76%) involving the auditory (neurosensory deafness, serous otitis media, and malformation of the external ear), respiratory (tracheomalacia), oro-gastrointestinal (cleft palate, oronasal reflux and inguinal hernia), genitourinary (hydronephrosis), cardiovascular (atrial septal defect), musculoskeletal and visual systems;
(3) presence of a cartilage core;
(4) a scarcity of bilateral lesions (1 of 17);
(5) located in the middle or lower third of the neck; and
(6) increased prevalence anterior to the SCM muscle.^[2]^

CCBR can have either rod-shaped elastic or hyaline cartilage core surrounded by normal skin and subcutaneous tissues. They are located in the middle or lower third of the neck, anterior to or over the SCM muscle. The lesion presents at birth and has no or very slow growth. The lesion has no connection with deep structures but adherence to the fascia of the SCM muscle is often reported. There is no report of underlying sinuses and cysts.^[12]^ Ultrasonography can be useful for describing the lesions, which have the characteristic presence of a tubular cartilage that extends to the SCM muscle. CCBR is often associated with cardiac or genitourinary abnormalities, which have been reported in 11% to 76% of cases.^[13]^ Therefore, preoperative additional imaging studies of the abdomen and heart are recommended.

In our study, we examined the previous medical literatures and inferred that there was no correlation between anomalies on unilateral CCBR and bilateral CCBR (Table 1).

As treatment for CCBR and to obtain an accurate histologic diagnosis, complete surgical excision is recommended. If the patient has problems with tolerating anesthesia, the excision can be postponed.

Tarmir et al suggested surgical treatment of CCBR just before starting school, which allows minimization of surgical complications and spares the child of the psychological complications; however, it can be postponed to a suitable and safe age.^[14,15]^

## 5 Conclusions

Bilateral CCBR is a rare condition. CCBR should be included in the differential diagnosis for congenital neck lesions in pediatric patients. The treatment of choice of CCBR is complete surgical excision. Further, careful preoperative assessments are needed for investigating associated lethal anomalies by abdominal ultrasound and cardiac examination.

## Author contributions

**Conceptualization:** Taejin Park, Han Shin Lee.

**Data curation:** Taejin Park, Hyo Jung An, Han Shin Lee.

**Formal analysis:** Han Shin Lee.

**Investigation:** Tae Han Kim.

**Methodology:** Jae Yool Jang, Jung Woo Woo.

**Project administration:** JinKwon Lee.

**Resources:** Sang Ho Jeoung.

**Supervision:** Eun Jung Jung.

**Validation:** Han Shin Lee.

**Visualization:** Han Shin Lee.

**Writing – original draft:** Han Shin Lee.

**Writing – review & editing:** Taejin Park.
